# 

**Published:** 1847-08

**Authors:** 


### CONTENTS.

### PART I.—ORIGINAL COMMUNICATIONS.

ART. 1. Extirpation of an Abdominal Tumor of Large Size. By D. Brainard, M D., Professor, &c., &c.
ART. 2. The Pathology and Treatment of Periodical Fevers. Read before the Rock River Medical Society. By S. G. Armor, M.D.
ART. 3. Observations on Milk Sickness. By Dr. J. H. McNutt.
ART. 4. Inflammation of the Brain. By G. N. Fitch, M.D., Prof., &c., &c.
ART. 5. Inquiries and Objections in regard to some parts of a Communication from Dr. Henry on the Treatment of Fevers and Inflammations without Mercury. By Dr. U. P. Golliday.
ART. 6. Case of Rupture of the Uterus—Recovery. By Dr. John S. Davis.
ART. 7. Remarks upon the Organization of the Medical Department of the Army and the Effects of Marching and a Camp Life in producing and modifying Disease. By W. B. Herrick, M.D., Prof., &c., &c.

### PART II.—REVIEWS.

ART. 8. Encyclopedia Americana: Supplementary Volume. A Popular Dictionary of Arts, Sciences, &c. Edited by Henry Vethake, L.L.D. Prof., &c.

### PART III.—BIBLIOGRAPHICAL NOTICES.

ART. 9. A Practical Treatise on the Diseases of Children. By D. Francis Condie, M.D., &c., &c.
ART. 10. The Hand Book of Human Anatomy, General, Special, and Topographical. Translated from the German of Alfred Von Behr. By John Birkett, &c., &c.
ART. 11. The Medical Students' Vade Mecum, or Manual of Examinations, &c., &c. By Geo. Mendenhall, M.D., &c., &c.
ART. 12. Medical Botany, &c. By R. Eaglesfield Griffith M.D., &c., &c.
ART. 13. A Practical Treatise on Inflammation, Ulceration, and Induration of the neck of the Uterus, &c., &c. By Jas. Henry Bennett, M.D., &c., &c.
ART. 14. The Half-Yearly Abstract of the Medical Sciences. Edited by W. H. Ranking, M.D., &c., &c.
ART. 15. The American Journal of Medical Sciences. Edited by Isaac Hays, M.D., &c., &c.
ART. 16. The Medico-Chirurgical Review and Journal of Practical Medicine. Republished by R. & G. S. Wood, N. Y.

### PART IV.—SELECTIONS.

1. Claims of the Public upon Professional Benevolence.
2. Discovery of the Letheon.
3. Case of Ovariotomy.
4. Spontoneous Evolution.
5. Human Tripod.
6. Professor Simpson on Artificial Separation of the Placenta.
7. Quinine in Coffee.
8. A Case of Injury of the Eye.
9. Quackery and the Newspaper Press.
10. On the Use of Quinine.
11. On the Effects of Blisters on the Young Subject.
12. Inhalation of Nitrous Oxide.

### PART V.—EDITORIALS.

ART. 1. On the Organization of the Medical Profession.
ART. 2. Hydriodate of Potash in Acute Hydrocephalus.
ART. 3. Medical Library and Reading Room.
ART. 4. Private Hospital for the Insane at Chicago.
ART. 5. Accidents by Lightning.
ART 6. Selling a Practice.
ART. 7. St. Louis Medical and Surgical Journal.
ART. 8. Rush Medical College.
ART. 9. New Work on Obstetrics and Diseases of Women and Children.
ART. 10. Medical Reform.
ART. 11. Miscellaneous Intelligence.
ART. 12. Notice to Readers and Correspondents.

